# Anatomical Study of the Atrioventricular Nodal Branch of the Heart

**DOI:** 10.7759/cureus.35412

**Published:** 2023-02-24

**Authors:** Joe Iwanaga, Sreeganesh Manoharan, Juan J Cardona, Samir Anadkat, Tsuyoshi Saga, Marios Loukas, R. Shane Tubbs

**Affiliations:** 1 Department of Neurosurgery, Tulane University School of Medicine, New Orleans, USA; 2 Department of Neurology, Tulane University School of Medicine, New Orleans, USA; 3 Department of Anatomy, Division of Gross and Clinical Anatomy, Kurume University School of Medicine, Kurume, JPN; 4 Department of Medicine, Tulane University School of Medicine, New Orleans, USA; 5 Department of Structural and Cellular Biology, Tulane University School of Medicine, New Orleans, USA; 6 Department of Anatomical Sciences, St. George's University, St. George, GRD; 7 Department of Surgery, Tulane University School of Medicine, New Orleans, USA; 8 Department of Neurosurgery, Ochsner Neuroscience Institute, Ochsner Health System, New Orleans, USA

**Keywords:** cadaver, sinoatrial node, atrioventricular node, coronary artery, heart

## Abstract

Background

The atrioventricular (AV) node is a relay station for electrical signals passing between the atria and ventricles. The artery supplying the AV node is functionally important, and its anatomical topography is relevant during invasive procedures. Therefore, the aim of this study was to identify and understand the variations of the origin of the AV nodal branch (AVNb) and its variations.

Materials and methods

We dissected 31 adult human hearts to evaluate their AVNb and its variations. A classification scheme was used to detail the morphology found for each of these arteries.

Results

We identified five distinct origins of the AVNb: AVNb originating from the right coronary artery (RCA) proximal to the inferior interventricular branch (IVb) (type I, 3.2%), AVNb originating from the junction of the RCA and IVb (type II, 19.4%), AVNb originating from the RCA distal to the IVb (type III, 64.5%), AVNb originating from the IVb (type IV, 6.5%), and AVNb originating from the circumflex branch of the left coronary artery (LCA) (type V, 6.5%).

Conclusions

Our study provides data on the morphology and variations of the AVNb. Such information can assist in better diagnoses based on imaging, better guide invasive procedures, and provide the cardiac surgeon with an improved method of classifying the AVNb and its branches during procedures of the coronary arteries and their branches.

## Introduction

A network of specialized muscle cells sends electrical signals to the rest of the heart muscle causing a contraction, i.e., the cardiac conduction system (CCS). The main parts of the system consist of the sinoatrial (SA) node, atrioventricular (AV) node, bundle of His, right and left bundles, and Purkinje fibers. A signal through the CCS starts with the SA node by causing the atrial muscles to contract. Then, the signal travels to the AV node, through the bundle of His, down the right and left bundles, and via Purkinje fibers, causing the ventricles to contract [1].

The SA node is a crescent-shaped structure, 3.3-6.7 mm wide, 7.3-29.5 mm long, and 1.0-2.2 mm deep, located at the embryonic junction between the parts derived from the atrium proper and the right atrium’s venous part [1-7]. Thus, the SA node is located relatively superficial in the heart [8,9]. The AV node is part of the conduction system of the heart [10], which is located within the triangle of Koch bordered by the septal leaflet of the right atrioventricular valve anteriorly and the tendon of Todaro posteriorly [11-13]. Identifying the variations in the blood supply to the AV node is relevant in not only clinical procedures but also surgical ones involving the coronary arteries [14]. Traditionally, the atrioventricular nodal branch (AVNb) has been reported to be located at the crux cordis, originating either from the right coronary artery (RCA) or branches off of the left coronary artery (LCA)/circumflex branch of the LCA (LCxA) and continuing into the triangle of the atrioventricular node (Koch’s triangle) [15,16]. According to Futami et al., the majority of AVNb run into the myocardium of the inferior interventricular sulcus and continue along the septal margin of the right AV valve toward the AV node, and in one case, the AVNb originated from the SN nodal branch and descended toward the AV node [10,17]. The vessel might also arise from the inferior interventricular branch (IVb) of the RCA [18]. The aim of this study was to investigate the origin of the AVNb and describe its morphology to provide a better clinical and surgical understanding of the blood supply to the region of the AV node.

This article was previously presented as a meeting abstract at the 2022 American Association of Clinical Anatomists (AACA) 39th Annual Meeting in Fort Worth, Texas, on June 13-17, 2022.

## Materials and methods

**Figure 1:**
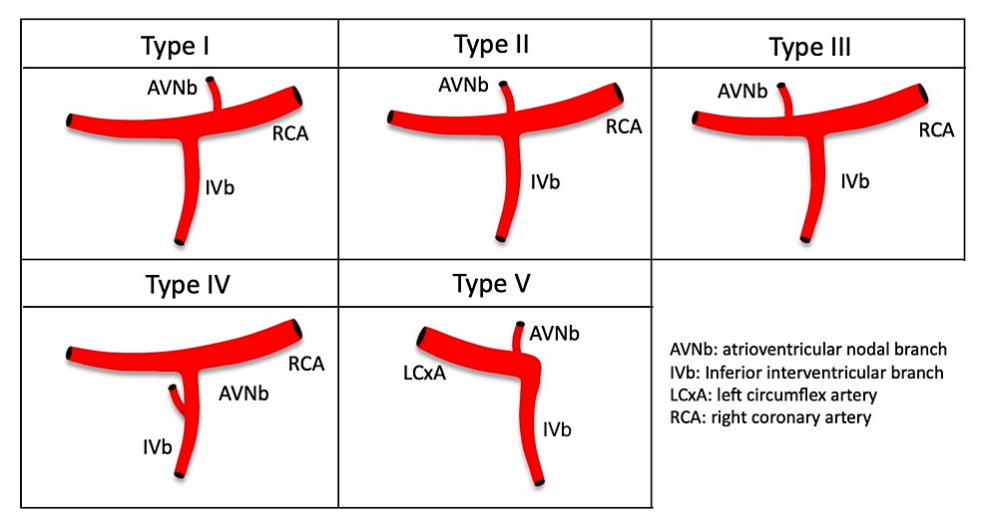
Classification of the origin of the atrioventricular nodal branch Type I: AVNb originating from the RCA proximal to the IVb, type II: AVNb originating from the junction of the RCA and IVb, type III: AVNb originating from the RCA distal to the IVb, type IV: AVNb originating from the IVb, type V: AVNb originating from the LCxA AVNb: atrioventricular nodal branch, IVb: interventricular branch, LCxA: circumflex branch of the LCA, RCA: right coronary artery

Thirty-one formalin-fixed adult human hearts from the Tulane University School of Medicine were used. The specimens were derived from 16 female and 15 male cadavers. The age at death of the specimens ranged from 55 to 100 years with a mean of 81.7 years. Specimens found to have surgical scars (e.g., coronary artery bypass grafting) over the thorax were excluded. Both the right and left coronary arteries and the branches were dissected carefully to identify the branch in the inferior pyramidal space. When the arterial branch running through the inferior pyramidal space was found, it was traced to confirm that it did not end in the myocardium. The origin of the AV nodal branch was documented and classified based on the results as depicted in Figure 1.

The diameter of the AVNb and coronary artery (either the RCA or LCA where the AVNb originated) was measured at their origin from the ascending aorta. Two anatomists (JI and RST) observed all the specimens and formulated the types. All measurements were performed using microcalipers (Mitutoyo, Kawasaki, Japan). Statistical analysis between diameter and sex was conducted using an unpaired t-test with significance set at p<0.05.

The present study protocol did not require approval by the ethics committees in our institutions and was performed in accordance with the requirements of the Declaration of Helsinki (64th World Medical Association (WMA) General Assembly, Fortaleza, Brazil; October 2013). Every effort was made to follow all local and international ethical guidelines and laws that pertain to the use of human cadaveric donors in anatomical research [19].

## Results

The diameter at the origin of the AVNb ranged from 0.88 to 2.42 mm with a mean of 1.58 mm (Table 1).

**Table 1:** Diameter of the AVNb and RCA/LCA AVNb: atrioventricular nodal branch, RCA: right coronary artery, LCA: left coronary artery, SD: standard deviation

		Mean (mm)	SD (mm)	
AVNb	Male	1.574	0.39	p=0.94
	Female	1.585	0.45	
RCA/LCA	Male	6.85	1.04	p=0.09
	Female	7.69	1.51	

**Figure 2:**
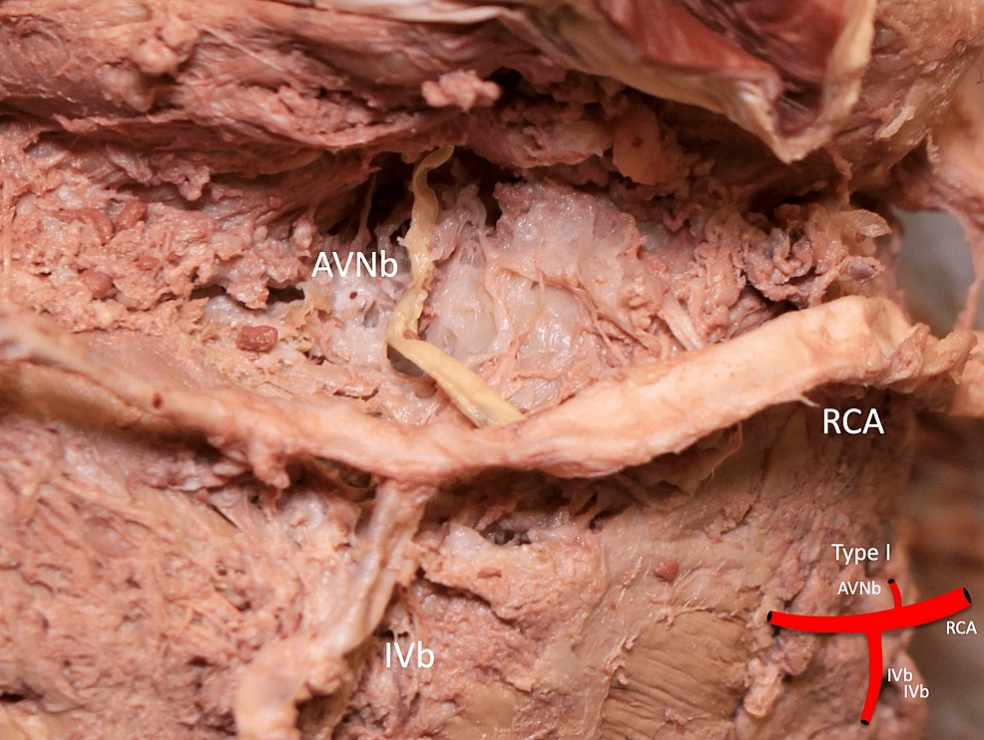
Type I: the AVNb is seen originating from the RCA proximal to the IVb AVNb: atrioventricular nodal branch, RCA: right coronary artery, IVb: inferior interventricular branch Scale bar: 10 mm

**Figure 3:**
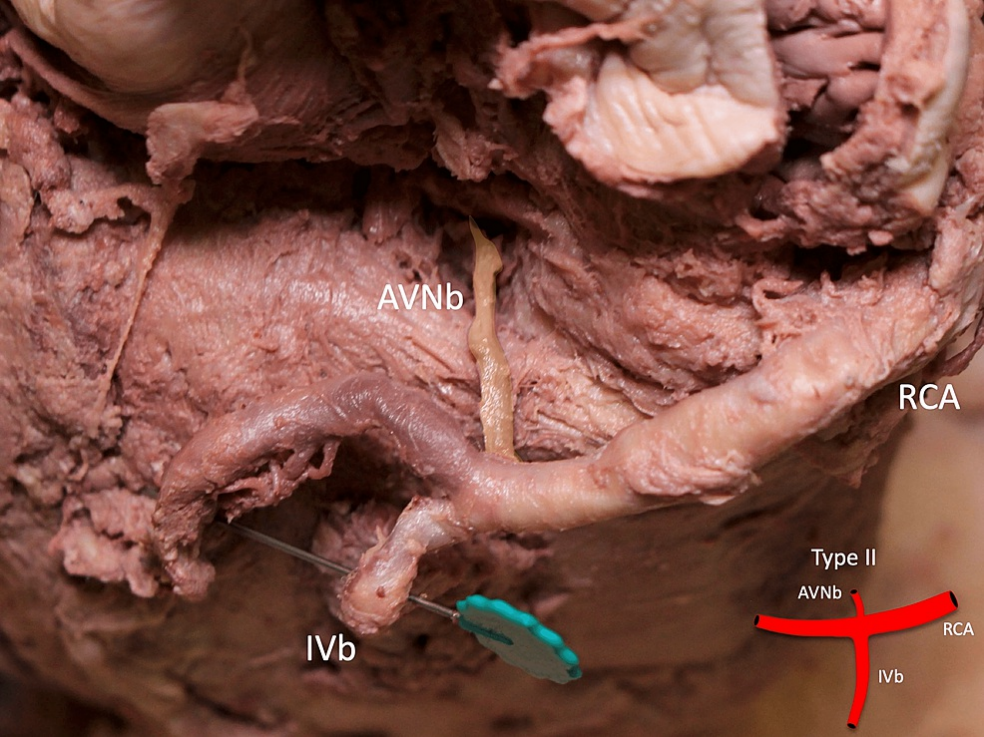
Type II: the AVNb is seen originating from the junction of the RCA and its IVb AVNb: atrioventricular nodal branch, RCA: right coronary artery, IVb: inferior interventricular branch Scale bar: 10 mm

**Figure 4:**
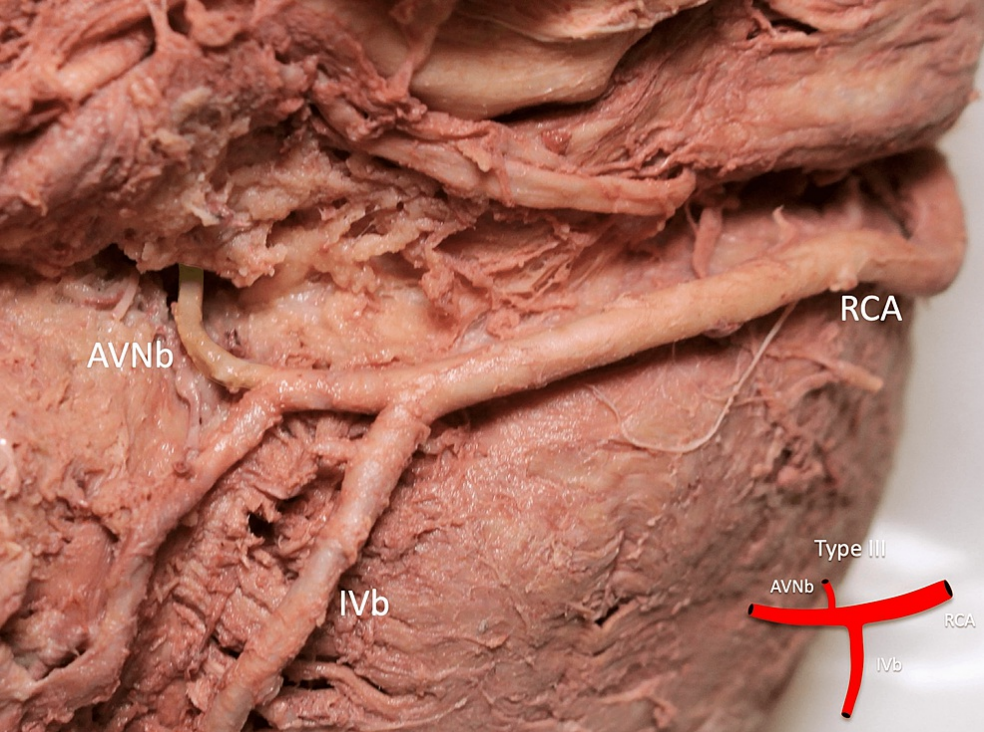
Type III: the AVNb is seen originating from the RCA distal to the IVb AVNb: atrioventricular nodal branch, RCA: right coronary artery, IVb: inferior interventricular branch Scale bar: 10 mm

**Figure 5:**
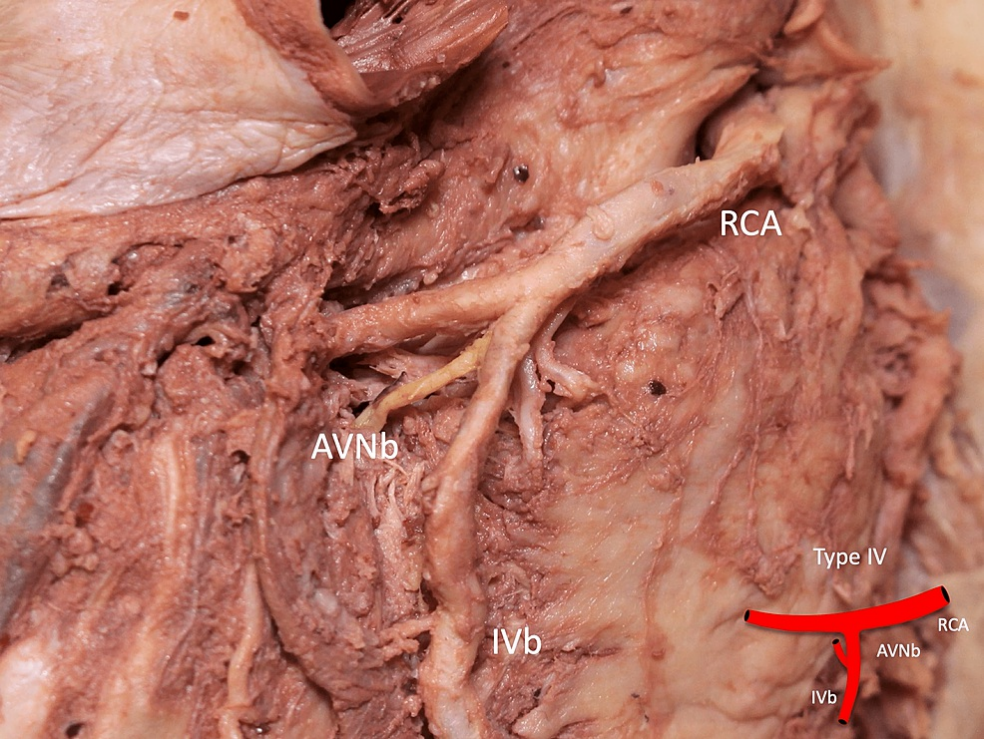
Type IV: the AVNb is seen originating from the IVb AVNb: atrioventricular nodal branch, RCA: right coronary artery, IVb: inferior interventricular branch Scale bar: 10 mm

**Figure 6:**
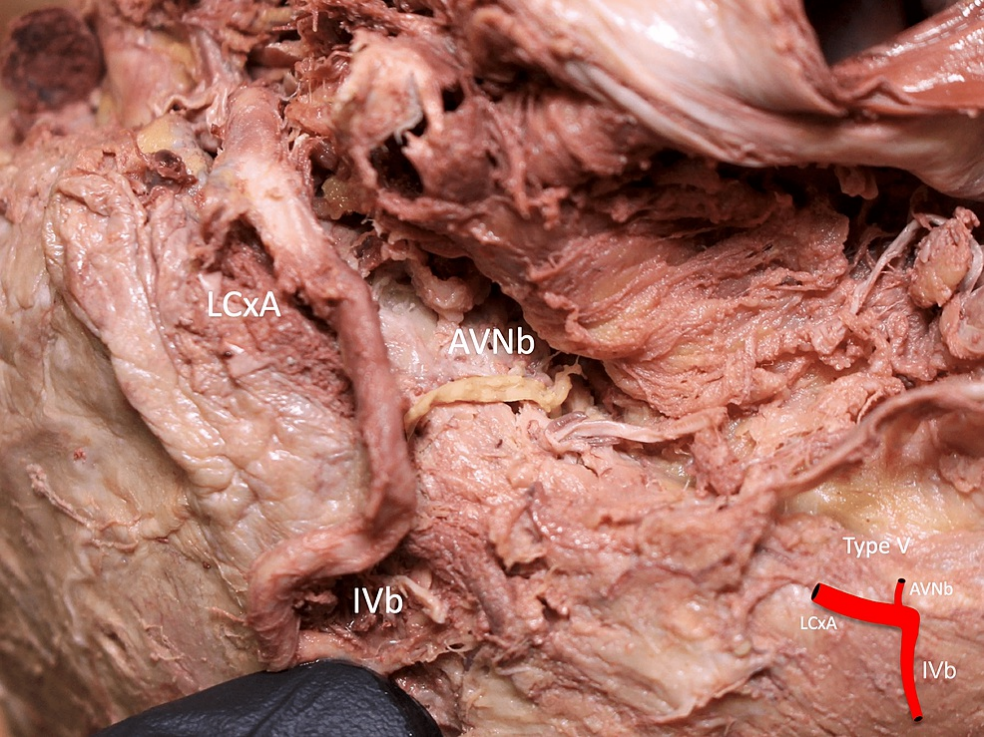
Type V: the AVNb is seen originating from the LCxA AVNb: atrioventricular nodal branch, LCxA: circumflex branch of the left coronary artery, IVb: inferior interventricular branch Scale bar: 10 mm

The diameter of the ipsilateral coronary artery ranged from 5.15 to 11.46 mm with a mean of 7.29 mm. Type I (Figure 2), type II (Figure 3), type III (Figure 4), type IV (Figure 5), and type V (Figure 6) were found in one (3.2%), six (19.4%), 20 (64.5%), two (6.5%), and two (6.5%) specimens, respectively (Table 2).

**Table 2:** Types of the origin of the AVNb by gender AVNb: atrioventricular nodal branch

Type	Male	Female	Total
Type I	0	1	1
Type II	2	4	6
Type III	12	8	20
Type IV	0	2	2
Type V	1	1	2
	15	16	31

**Figure 7:**
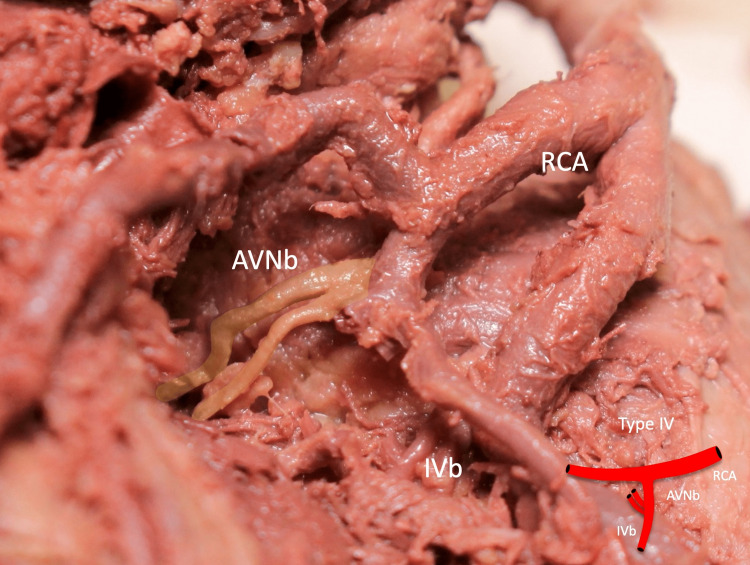
Duplicated AVNb seen as type IV AVNb: atrioventricular nodal branch, IVb: inferior interventricular branch, RCA: right coronary artery Scale bar: 10 mm

In one type IV specimen, duplication of the IVb was observed (Figure 7). Two hearts were left dominant, while the remaining hearts were right dominant.

**Figure 8:**
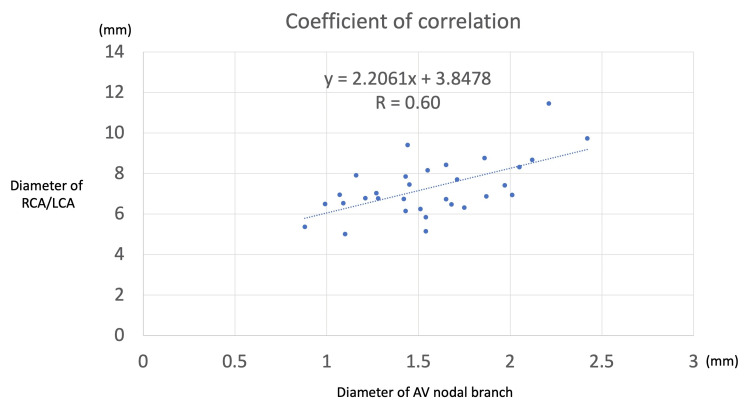
Coefficient of correlation between the diameters of the AVNb and RCA/LCA There was a moderate correlation (r=0.6). AVNb: atrioventricular nodal branch, LCA: left coronary artery, RCA: right coronary artery

Four specimens (all females and all right dominant) died of cardiac arrest. Of these, two were type III and two were type IV AVNb. The mean diameter of the AVNb and RCA was 1.41 mm and 6.80 mm, respectively. There were no statistically significant differences in the diameter of either the AVNb or the RCA/LCA between males and females. The coefficient of correlation between the diameter of the AVNb and the coronary artery of origin was 0.60 (moderate correlation) (Figure 8). No major anatomical variations of the coronary arterial circulation were noted on any heart.

## Discussion

The AVNb is most often a branch of the right coronary artery in approximately 90% of cases entering into the triangle of Koch [20,21]. In some cases, the AVNb is a terminal branch of the LCxA, and in others, both coronary arteries supply the AV node [22]. For the origin of the AVNb, we classified our specimens as AVNb originating from the RCA proximal to the IVb (type I), AVNb originating from the junction of the RCA and IVb (type II), AVNb originating from the RCA distal to the IVb (type III), AVNb originating from the IVb (type IV), and AVNb originating from the LCxA (type V) [18,23].

The variations in the origin of the coronary arteries can be attributed to the embryological development of the heart. Specifically, cells of the sinus venosus join the muscular layer of the heart to continue its development [24]. From this stage, the sinus venosus forms a blood vessel plexus, which eventually remodels into the arteries and veins of the heart [25]. This plexus is preferentially developed from a group of blood islands near the epicardium of the interventricular sulcus near the apex of the heart. After the blood islands are formed, there is a rudimentary venous connection that exists between the coronary sinus and this plexus. Following the next step in coronary vasculature development, the arterial system is established between the epicardial plexus and the ostia in the aortic sinuses [26]. The vascular connection from the blood islands eventually forms what we know as the branches off of the RCA and LCA.

Our understanding of AV node anatomy and function is important in clinical medicine. The AV node plays a crucial role in mediating the signal between the atria and ventricles; this helps regulate premature contractions before the complete filling of the ventricles. The AV node’s decremental conduction is a valuable feature that allows for the slowing of conduction from the SA node as well as random inputs into the AV node itself. Additionally, it can function as the primary pacemaker of the heart during times of SA node dysfunction or failure [27].

The AVNb originated from the RCA distal to the IVb in approximately 65% of our specimens (type III). The anatomical importance of the left AVNb is also important in, for example, surgical manipulation of the right fibrous ring [22,28]. Damage to the AV node and/or the AVNb has been shown to produce pathological conditions related to the pacing of the heart. Recent studies have shown that slow infusion of low concentrations of ethanol into the AVNb or targeted ablation of AV conduction can be a useful therapy for patients who have failed, for example, radiofrequency ablation or specifically those with drug-resistant tachycardia [29,30].

First-degree AV block is the most common pathophysiology seen with the conduction system of the AV node [31]. The presence of AV block demonstrates prolonged conduction from the atria to the ventricles, typically seen in increased vagal tone in younger patients and greater fibrotic changes in older patients [32]. In patients with nonatherosclerotic narrowing of the AVNb, it has been implicated as a cause of sudden cardiac arrest due to a dramatic decrease in blood supply to the musculature, as well as to the AV node [16]. The importance of the diameter of the AVNb might also play a role in a patient’s likelihood of experiencing sudden death [33]. Burke et al. found that of patients who died of sudden cardiac death, the mean diameter of the AVNb was 0.58±0.23 [33]. In our cadaveric study, the mean diameter of the AVNb was 1.56 mm with a range of 0.88-2.42 mm. Our classification system might also be useful to clinicians in discussing the anatomical variations of the AVNb. For example, catheterization of the AVNb for ablative procedures would be improved with a wider knowledge of the various morphologies of the AVNb as identified in our anatomical study.

Declaration

We sincerely thank those who donated their bodies to science so that anatomical research could be performed. Results from such research can potentially increase mankind’s overall knowledge that can then improve patient care. Therefore, these donors and their families deserve our highest gratitude [34].

## Conclusions

Based on our study, five variations of the AVNb’s origin were seen. An AVNb originating from the RCA distal to the IVb (type III) was more common than the other four variations, and the prevalence of this variation was approximately 65% in our specimens. In regard to the diameter of the coronary arteries, there were no statistically significant differences in diameter (of either the AVNb or the RCA/LCA) between males and females.

Our study provides data on the variations of the AVNb. Such information can assist in better diagnoses based on imaging, e.g., cystic tumors of the AV nodal region, and provide the cardiac surgeon with an improved method of classifying the AVNb and its branches during procedures of the coronary arteries and their branches.
